# Supplementary material for: Experimental validation of the mechanism of stomatal development diversification
Source: J Exp Bot. 2023 Aug 9;74(18):5667–81. doi: 10.1093/jxb/erad279 (PMC10540739; doi:10.1093/jxb/erad279)
Supplement: erad279_suppl_Supplementary_Figures_S1-S8_Table_S1 [file erad279_suppl_supplementary_figures_s1-s8_table_s1.pdf]

Supplementary information

Title: Experimental validation of the mechanism of stomatal development diversification

Authors: Yuki Doll, Hiroyuki Koga, and Hirokazu Tsukaya

Number of supplementary figures: 8

Number of supplementary tables: 1

Supplementary Figure S1

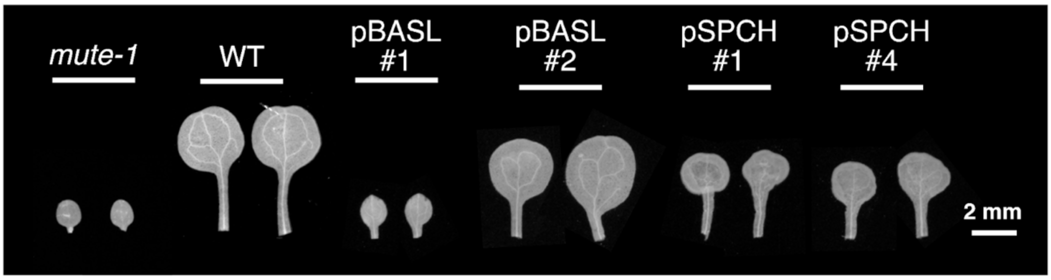

Supplementary Figure S1 Cotyledon morphology of the other established ‘early-MUTE’ lines.

These images supplement the content of Figure 2. Cotyledons at 20 DAS are shown. Note that both pSPCH and pBASL show unstable and severely retarded growth phenotypes.

Supplementary Figure S2

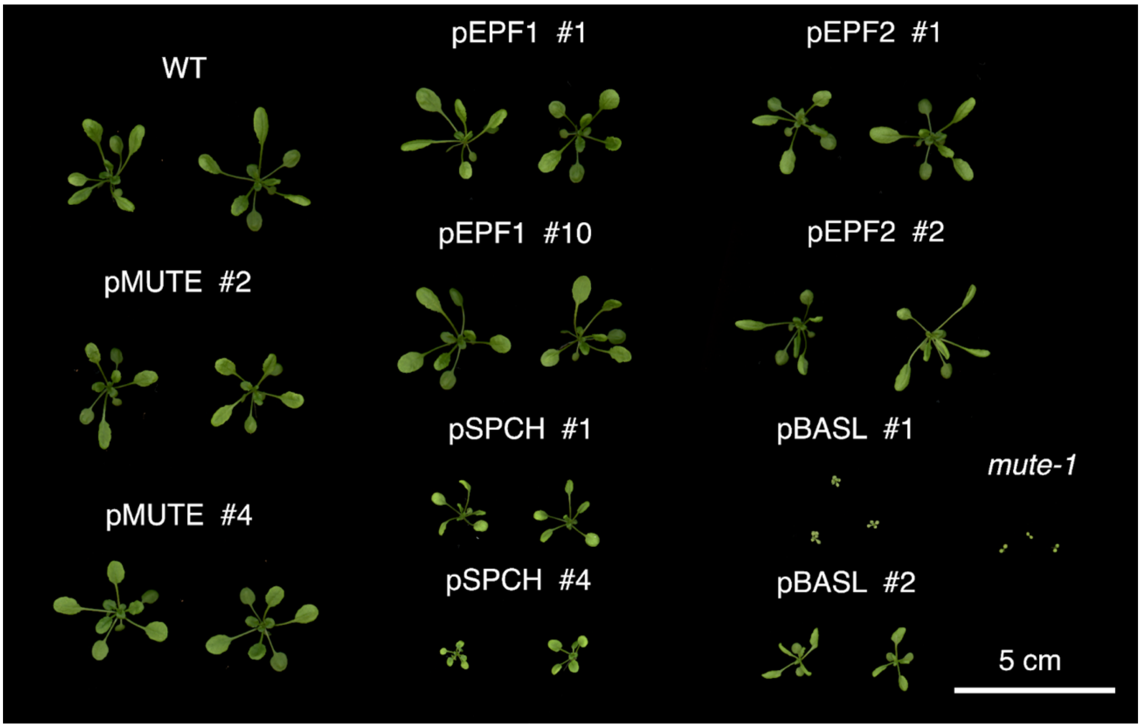

Supplementary Figure S2 Gross shoot morphology of the established lines.

Images depicting the shoot morphology of the lines shown in Figure 2 and Supplementary Figure S1.

Supplementary Figure S3

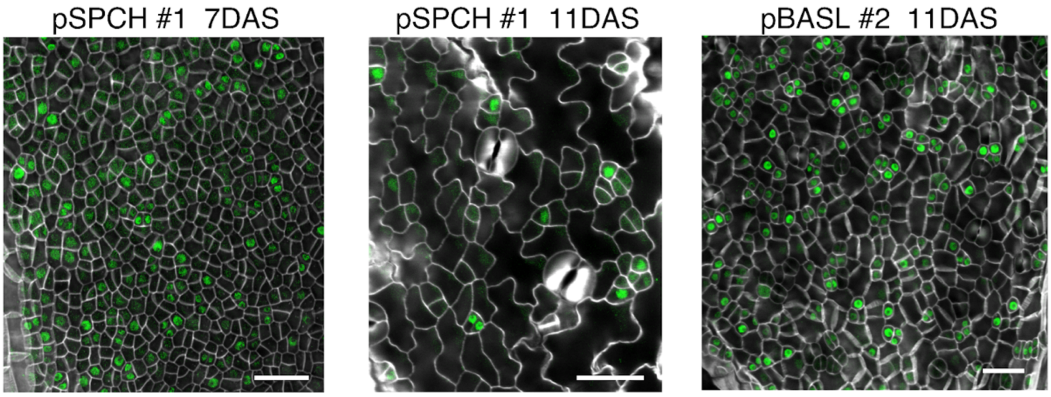

Supplementary Figure S3 Confocal microscopy images of the abaxial surface of developing first foliage leaves in the other early-MUTE lines.

Images for pSPCH #1 at 7 DAS, pSPCH #1 at 11 DAS, and pBASL #2 at 11 DAS are shown. The data support the images shown in Figure 3. The MUTE-GFP signal is shown in green, and the Calcofluor-stained cell wall is shown in gray. Bar: 25 μm.

Supplementary Figure S4

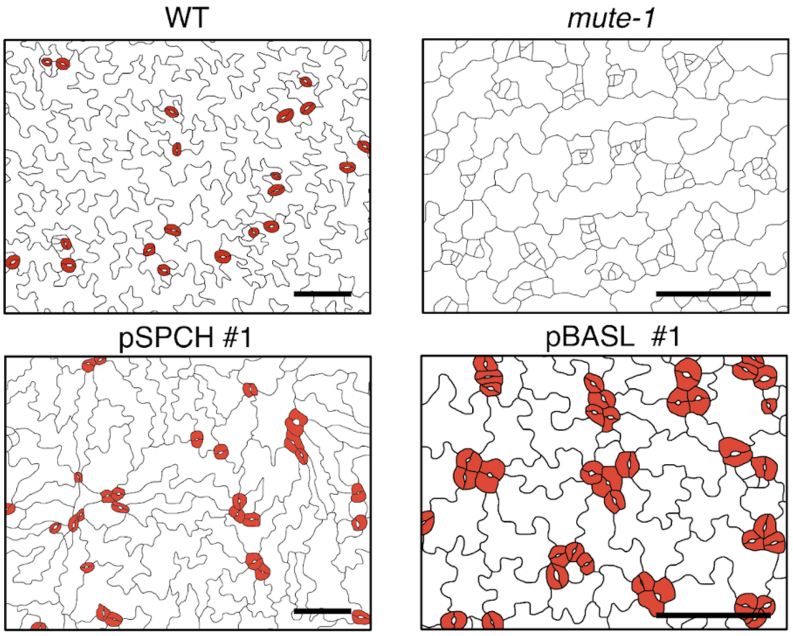

Supplementary Figure S4 Epidermal morphology of the abaxial surface of cotyledons in WT, *mute-1*, pSPCH #1, and pBASL #1.

These supplement the epidermal morphology images shown in Figure 4. The abaxial surface of 20-DAS cotyledons are shown. Bar: 100 μm.

Supplementary Figure S5

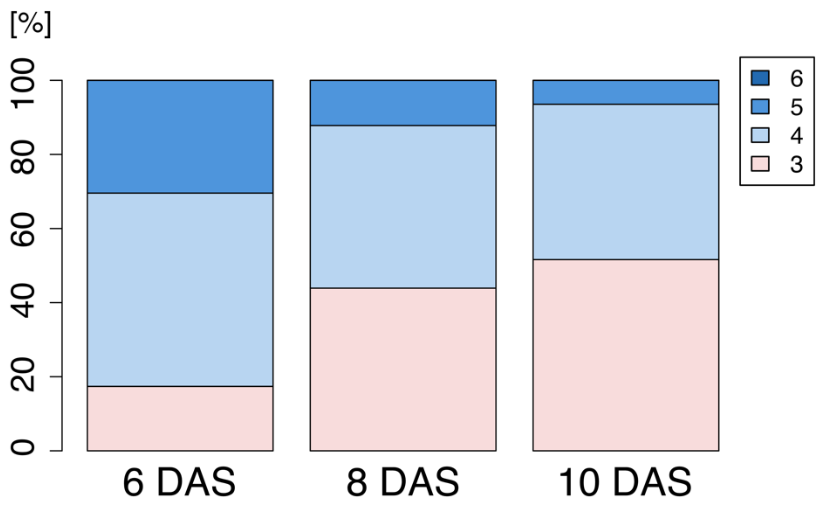

Supplementary Figure S5 Changes in the neighbor cell number of stomata during the course of foliage leaf development.

The number of neighboring cells of each stoma was counted in 6, 8, and 10 DAS foliage leaves (abaxial surface; the samples analyzed in Fig. 3) in pMUTE Arabidopsis. *n*=23, 41, and 31 stomata for 6, 8, and 10 DAS, respectively.

Supplementary Figure S6

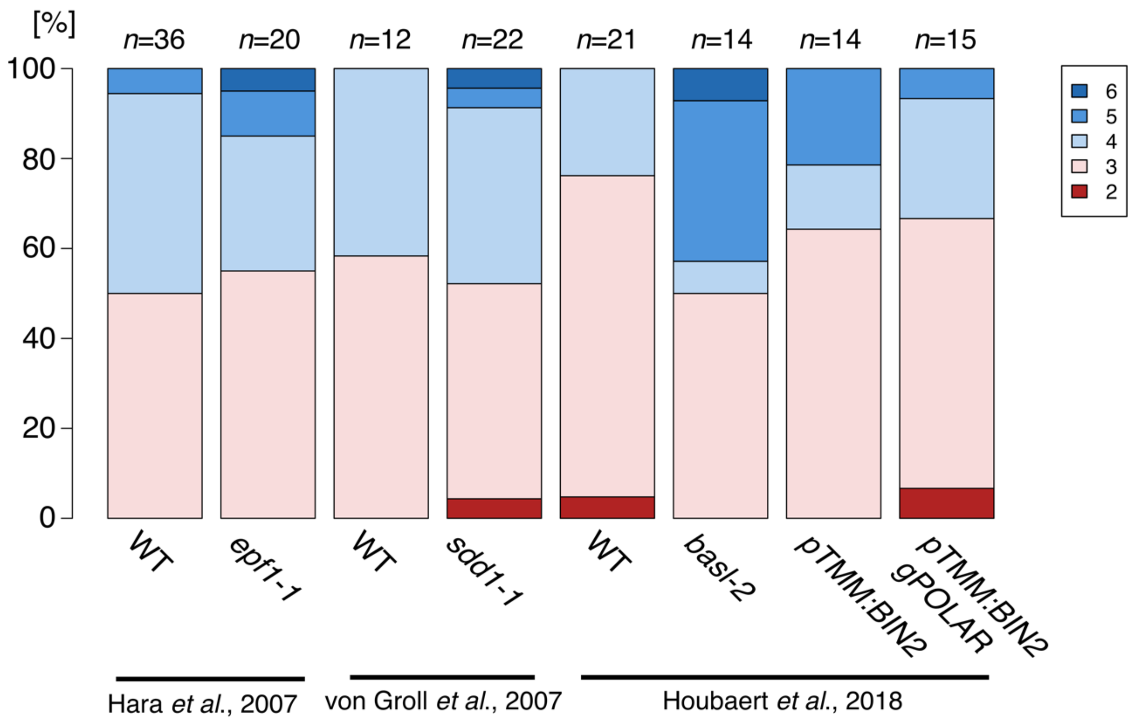

Supplementary Figure S6 Quantification of the number of neighboring cells of stomata in cluster-forming mutants.

The cell number was counted using the images in published papers (Hara *et al.*, 2007; Houbaert *et al.*, 2018; von Groll *et al.*, 2007). As shown in Fig. 4G, clustered stomata (60% for *epf1-1* and 15% for *sdd1-1*; 25% for *basl-2*; 10% for *pTMM:BIN2*; 42% for *pTMM:BIN2* / *gPOLAR*) were omitted from the analysis, and only unclustered stomata (40% for *epf1-1* and 85% for *sdd1-1*; 75% for *basl-2*; 90% for *pTMM:BIN2*; 58% for *pTMM:BIN2* / *gPOLAR*) were analyzed. The number of samples (*n*) is shown at the top of each graph.

Supplementary Figure S7

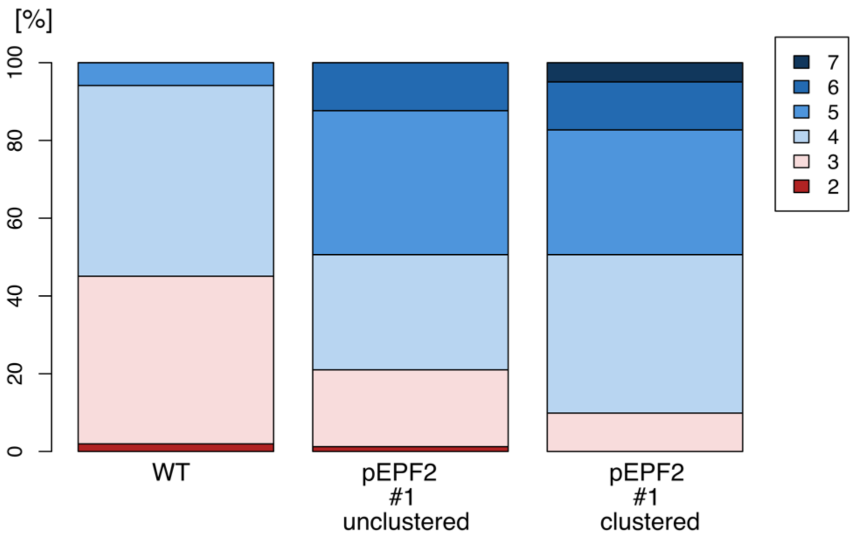

Supplementary Figure S7 Quantification of the number of neighboring cells of clustering stomata in pEPF2.

The number of neighboring cells for clustering stomata (*n*=81) in pEPF2 was counted and presented with the data of non-clustering stomata in pEPF2 and WT (Fig. 4G). Here, a neighboring stoma (a pair of guard cells) was counted as one neighboring cell.

Supplementary Figure S8

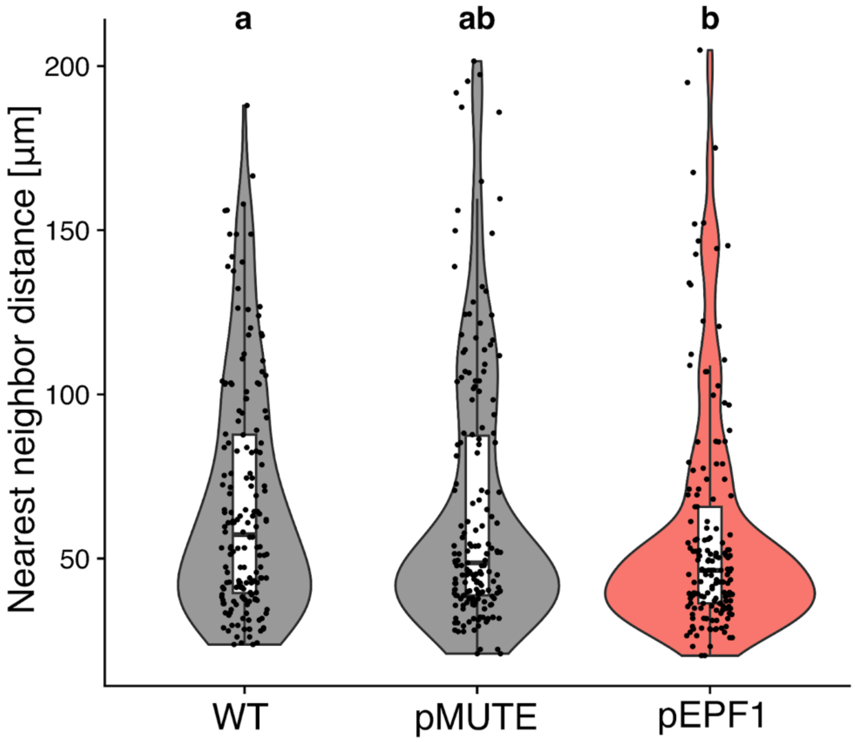

Supplementary Figure S8 Quantification of the distance of each stoma from its nearest neighbor.

More than 165 stomata from 10 mature cotyledons were analyzed for each line. Groups labeled with different letters are significantly different (P<0.05) according to the Brunner-Munzel test with Bonferroni correction.

Supplementary Table S1

Component	F/R	Sequence (5' → 3')	Reference / additional information
AtBASL promoter	F	ggccagtgccaagctGGATATTCATCTTTGCTACA	Dong *et al.*, 2009
	R	agcgatgtgagacatGGCTGTTATGTTTGTGT	
AtEPF2 promoter	F	ggccagtgccaagctTGGTCTAGAGAACAAGTGAA	Hara *et al.*, 2009
	R	agcgatgtgagacatGTTTATAATCTTTTTTTTTAACAAG	
AtEPF1 promoter	F	ggccagtgccaagctTGGTTATGAAATATTTTCCC	Hara *et al.*, 2007
	R	agcgatgtgagacatGATATATTATCGCAA	
AtSPCH promoter	F	ggccagtgccaagctTATGATCATTTGTAT	Pillitteri *et al.*, 2007
	R	CGTGATTAGAGATATATCCTTCTCTCTCTC	
AtMUTE CDS	F1	ATGTCTCACATCGCTGTTGAAAGGA	For pBASL, pEPF2, pEPF1
	F2	atatctctaatcacgATGTCTCACATCGCT	For pSPCH
	R	ATTGGTAGAGACGATCACTTCATCA	
GFP	F	atcgtctctaccaatGTGAGCAAGGGCGAG	
	R1	gatcggggaaattcgTTACTTGTACAGCTC	For pGWB backbone (pEPF1, pSPCH)
	R2	cttcatcttcataagTTACTTGTACAGCTC	For pRI backbone (pEPF2, pBASL)

Supplementary Table S1 List of the primers used for constructing vectors for transformation to Arabidopsis. The vectors for the establishment of pBASL and pEPF2 lines were constructed by using the pRI backbone, and the vectors for the establishment of pEPF1 and pSPCH lines were constructed by using the pGWB backbone. Letters in lowercase are sequences artificially added to the native Arabidopsis sequence for plasmid construction.

References

Dong J, MacAlister CA, Bergmann DC. 2009. BASL controls asymmetric cell division in Arabidopsis. Cell **137**, 1320–30.

Hara K, Kajita R, Torii KU, Bergmann DC, Kakimoto T. 2007. The secretory peptide gene *EPF1* enforces the stomatal one-cell-spacing rule. Genes & Development **21**, 1720–1725.

Hara K, Yokoo T, Kajita R, Onishi T, Yahata S, Peterson KM, Torii KU, Kakimoto T. 2009. Epidermal cell density is autoregulated via a secretory peptide, EPIDERMAL PATTERNING FACTOR 2 in Arabidopsis leaves. Plant & Cell Physiology **50**, 1019–1031.

Houbaert A, Zhang C, Tiwari M, *et al.* 2018. POLAR-guided signalling complex assembly and localization drive asymmetric cell division. Nature **563**, 574–578.

Pillitteri LJ, Sloan DB, Bogenschutz NL, Torii KU. 2007. Termination of asymmetric cell division and differentiation of stomata. Nature **445**, 501–505.

von Groll U, Berger D, Altmann T. 2002. The subtilisin-like serine protease SDD1 mediates cell-to-cell signaling during Arabidopsis stomatal development. The Plant Cell **14**, 1527–1539.
